# Supplementary material for: Loneliness and delusion-like experiences among women: Mediating role of procrastination and boredom proneness
Source: PLoS One. 2025 Aug 4;20(8):e0329607. doi: 10.1371/journal.pone.0329607 (PMC12321127; doi:10.1371/journal.pone.0329607)
Supplement: Attachment — Submitted filename: Response to Reviewers.docx [file pone.0329607.s002.docx]

The main change involved limiting the obtained results exclusively to women, which was highlighted by modifying the title, updating the theoretical section of the manuscript, and re-running all statistical analyses on the female subgroup. Additionally, the noted limitations regarding the data collection method and the low Cronbach’s alpha reliability coefficient for one of the instruments were appropriately justified in the paragraph describing the study’s limitations.

Response to Reviewer #1’s comments:
First, I would like to sincerely thank the reviewer for their valuable and constructive comments. The insights provided have been very helpful in improving the quality and clarity of the manuscript. I have carefully considered each point and addressed them as follows:

1) The sample was reduced exclusively to the group of women; the limitation of data collection via electronic means as a common method was justified in the study’s limitations section.

2) The reliability of the BPS instrument changed after reducing the sample size; moreover, this aspect was addressed in the limitations section with appropriate explanations.

3) Prior to conducting the mediation analysis, the variables were checked for multicollinearity criteria (VIF and Tolerance coefficients); the results did not indicate any multicollinearity issues. An explanation has been added in the “Results” section.

4) I am aware that this was not an experimental study, so causal relationships cannot be inferred. The direction of the relationships was based on theoretical and empirical assumptions presented in the theoretical section. Consequently, all conclusions were stated probabilistically. The penultimate paragraph of the discussion acknowledges the impossibility of claiming causality, which would require further experimental or longitudinal studies.

5) The lack of data on psychiatric history or current mental health status of participants was described in the study limitations.

6) The data have been made publicly available via the OSF platform (<https://osf.io/smky9/?view_only=339a37dac63a490eb54ccc0424c7962d>).

Response to Reviewer #2’s comments:
I would like to sincerely thank Reviewer #2 for thorough and insightful feedback. The constructive suggestions provided have greatly contributed to enhancing the manuscript. Below, I address each of the points raised:

1) The novelty of my study is the specific configuration of variables examined, which has not been previously investigated. In particular, the mediating role of procrastination and boredom proneness has not been studied in relation between loneliness and delusion-like experiences before. Moreover, this configuration has not been explored specifically in relation to women, which adds a unique perspective and contribution to the existing literature.

2) I have added a reference to a study that specifically addresses the experience of loneliness within both collectivistic and individualistic cultural contexts.

3) Under each hypothesis, paragraphs with theoretical assumptions and previous research findings that supported the hypotheses formulated in this article were included. In my opinion, this was done clearly.

4) I added a table with the demographic characteristics of the sample, and updated the description of the sample as well as the participant recruitment procedure in the methods section.

5) The text was edited and reviewed by a professional translation agency.

6) The study was limited exclusively to the group of women. The discussion section was updated accordingly.

7) DOI numbers were added to the missing references (some did not have identifiers). The sections on study limitations and future research directions were separated into distinct subsections.
